# Localization Based on MAP and PSO for Drifting-Restricted Underwater Acoustic Sensor Networks

**DOI:** 10.3390/s19010071

**Published:** 2018-12-25

**Authors:** Keyong Hu, Xianglin Song, Zhongwei Sun, Hanjiang Luo, Zhongwen Guo

**Affiliations:** 1 School of Information and Control Engineering, Qingdao University of Technology, Qingdao 266520, China; hukeyong@qut.edu.cn (K.H.); songxianglin@qut.edu.cn (X.S.); 2 School of Computer Science and Engineering, Shandong University of Science and Technology, Qingdao 266590, China; hjluo@sdust.edu.cn; 3 Department of Computer Science and Technology, Ocean University of China, Qingdao 266100, China; guozhw@ouc.edu.cn

**Keywords:** underwater acoustic sensor networks, beacon-free localization, maximum a posteriori, particle swarm optimization

## Abstract

Localization is a critical issue for Underwater Acoustic Sensor Networks (UASNs). Existing localization algorithms mainly focus on localizing unknown nodes (location-unaware) by measuring their distances to beacon nodes (location-aware), whereas ignoring additional challenges posed by harsh underwater environments. Especially, underwater nodes move constantly with ocean currents and measurement noises vary with distances. In this paper, we consider a special drifting-restricted UASN and propose a novel beacon-free algorithm, called MAP-PSO. It consists of two steps: MAP estimation and PSO localization. In MAP estimation, we analyze nodes’ mobility patterns, which provide the priori knowledge for localization, and characterize distance measurements under the assumption of additive and multiplicative noises, which serve as the likelihood information for localization. Then the priori and likelihood information are fused to derive the localization objective function. In PSO localization, a swarm of particles are used to search the best location solution from local and global views simultaneously. Moreover, we eliminate the localization ambiguity using a novel reference selection mechanism and improve the convergence speed using a bound constraint mechanism. In the simulations, we evaluate the performance of the proposed algorithm under different settings and determine the optimal values for tunable parameters. The results show that our algorithm outperforms the benchmark method with high localization accuracy and low energy consumption.

## 1. Introduction

Underwater Acoustic Sensor Networks (UASNs) have been widely applied in many fields such as underwater surveillance, pollution detection and disaster prevention [1,2]. Generally, an UASN is comprised of different types of nodes, which can be floating sensor nodes, surface buoys, Autonomous Underwater Vehicles (AUVs) and other application specific devices [3,4]. These nodes communicate with each other and sense underwater environments collaboratively. The sensed data is then analyzed to provide decision support for the upper applications. In this process, the locations of nodes need to be aware to interpret the sensed data meaningfully. Hence, localization is one of the critical services in UASNs.

Generally, an UASN consists of two types of nodes: beacon nodes and unknown nodes. The locations of beacon nodes are known in advance using GPS module or fixed deployment. They are powerful and can emit beacon signals to help unknown nodes’ localization. In contrast, unknown nodes are relatively cheap with limited energy and computation ability. The localization procedure aims to obtain the ranging measurements between unknown nodes and beacon nodes, and figure out the locations of unknown nodes from these measurements using specific localization algorithms. Therefore, the localization performance significantly depends on the ranging accuracy and the algorithm efficiency.

In UASNs, the ranging methods mainly include Time of Arrival (TOA), Time Difference of Arrival, Angle of Arrival (AOA) and Received Signal Strength Indicator (RSSI). Since underwater acoustic channel is time-varying, accurate attenuation models do not exist and thus RSSI method is rarely used [5]. AOA method requires multiple directional antennas, resulting in additional hardware costs [6]. TDOA method measures the time differences of the signal arrivals from beacon node pairs, which does not require time synchronization. However, uncertain Multiple Access Control (MAC) delays may incur large errors [6]. In contrast, TOA method is preferred in UASN localization. It estimates the distances using the difference of packet sending and receiving time, but requires time synchronization between sending and receiving nodes. Alternatively, two-way TOA [7] can eliminate the clock offset between two nodes and improve the ranging accuracy significantly.

In addition, the harsh underwater environments have a great impact on the accuracy of the TOA method. First, the signals can arrive from line of sight (LOS) or non-line of sight (NLOS) communication links due to the existence of reflective obstacles (e.g., sea surface, rocks and marine animals) [8]. The LOS signals do not always have strongest energy since underwater acoustic channel is time-varying and frequency selective [4]. Consequently, NLOS signals may be mistaken for LOS signals, which degrades the accuracy of the TOA method. Second, the acoustic speed varies with depth, temperature and salinity, known as stratification effect [9], resulting in that the signals propagate along a non-straight line. Hence, simply multiplying time-of-flight (TOF) with constant acoustic speed may introduce large errors to distance estimations. Most existing works neglect the multipath and stratification effects, or assume the incurred errors follows Gaussian distributions with fixed expectation and variance. However, this assumption does not hold true because the TOF error often increases with the distance between two nodes [10,11].

Another important factor on localization performance is the network topology. Due to the fluid property of underwater environment, nodes usually drift with currents and tides. Consequently, the network topology may change continuously. According to the degree of the restriction of nodes’ movements, there exist two drifting modes, which are free drifting and restricted drifting, respectively. For free drifting mode, nodes move without restrictions [6,9,12,13,14,15,16,17,18,19,20]. However, the complete randomness of nodes’ movements rarely provides useful information for localization. The a priori localization information can only be provided by designated beacon nodes. Consequently, the number of beacon nodes grows with the network scale and the network cost increases accordingly. For restricted drifting mode, a portion of nodes (beacon nodes, in most cases) move along restricted paths [3,21,22,23,24,25,26]. Nodes send beacon signals at specific points of their paths. Using the geometrical relations between the sending points and unknown nodes’ positions, the whole localization process only needs few beacon nodes, or even without the presence of beacon nodes. Therefore, the network cost can be reduced significantly.

Aiming at the above mentioned problems, this paper proposes a new localization method based on Maximum a Posteriori (MAP) estimation and Particle Swarm Optimization (PSO) techniques. Considering a portion of nodes may move out of the surveillance area due to free drifting, which leads to insufficient network coverage and increases the difficulties of network recycling and maintenance, we follow a Drifting-Restricted Underwater Acoustic Sensor Network (DR-UASN) [21,22], in which nodes are linked to fixed points by cables and each node’s movement is confined in a local hemisphere area. The motivation behind our method is to fully explore the priori localization information hidden in this special network for improving localization performance without the presence of beacon nodes. The whole localization process consists of two steps: MAP estimation step and PSO localization step. In the former step, the nodes that can communicate with each other form a cluster. The distances between cluster nodes are estimated using the TOA method. Then, the priori localization information and the distance measurements are fused to obtain the posterior probability distribution of unknown nodes’ locations and derive the weighted objective function by maximizing the logarithm of posterior distribution under the Bayesian framework. In the latter step, a swarm of particles are initialized according to its movement area, and then updated iteratively towards local and global best solutions with a certain speed. By calculating the fitness value of the objective function and communicating with each other, the particles collaboratively determine best location solution. Specifically, our contributions are mainly as follows:

- This paper proposes a novel localization method without the presence of beacon nodes for DR-UASNs, which achieves higher localization accuracy and lower computational cost compared with the benchmark method.
- The noises varying with distances are taken into account, which is modeled by additive and multiplicative noises. Hence the noises in distance measurements can be efficiently filtered for improving localization accuracy.
- The reference selection and bound constraint mechanisms are proposed to combat the problems of localization ambiguity and low convergence speed in the PSO step.

The rest of this paper is organized as follows: in Section 2, we briefly review existing works on UASN localization. Then, the network model is presented and the localization problem is formulated in Section 3. Section 4 presents the localization process of the MAP-PSO algorithm. In Section 5, we evaluate the performance of the MAP-PSO algorithm under different settings and conduct comparison with the AFLA algorithm. Finally, conclusions are drawn in Section 6.

## 2. Related Work

Existing localization schemes can be divided into two categories: range-free and range-based. While range-free schemes provide coarse-grained location estimations with low communication cost, range-based schemes can achieve a relatively high localization accuracy, but with additional communication and hardware cost. In this work, we are interested in range-based schemes. Next, we briefly review some works related to our method, and more detailed research review can be found in [4,27].

In general, range-based localization consists of two stages: distance estimation and location calculation. The ranging method commonly used is TOA, which gets the difference between packet sending and receiving time and estimates the distance by multiplying the time difference and the acoustic speed. However, it suffers from low accuracy due to multiple factors such as time synchronization, multipath effect and stratification effect. In [3,25] , the localization does not require time synchronization. It is assumed that beacon nodes move in the vertical direction and unknown nodes are stationary, which are unpractical in underwater environments. Several works jointly consider the time synchronization and localization problems [6,9,19,28], in which time synchronization is first performed to obtain clock skew and offset, and then the locations are estimated based on synchronized distance measurements. In [10], a novel ranging method is proposed under the assumption of isogradient sound speed profile (SSP), i.e., the sound speed is linearly related to the depth in each SSP layer. Given the depths of two nodes and the TOA measurements, it calculates the horizontal distance through a root finding algorithm. This method has high accuracy, but with high computational cost. Further, RAR [29] is proposed to enable real-time localization based on Bellhop model. Its main drawback is that the Bellhop model can’t reflect time-varying characteristic. In [8], TOA measurements from multiple paths are assumed to follow a mixture of three Gaussian distributions corresponding to LOS, SNLOS and ONLOS links, and then an EM algorithm is used to accurately classify different types of links.

In a 3D UASN, the locations of unknown nodes can be figured out by using classic multilateration algorithm and the ranging measurements to at least four beacon nodes. Further, the number of beacon nodes can be reduced to three by projecting them onto unknown node’s horizontal plane [7,12]. In [30], a hyperbola-based localization method is proposed, in which the ambiguity existing in multilateration can be eliminated. For large-scale and sparse UASNs, many unknown nodes can’t be localized due to lack of sufficient beacon nodes. Hence, an iterative localization strategy is commonly adopted to improve the localization coverage [15,25,31,32], in which unknown nodes that have been localized with high accuracy can be regarded as reference nodes to help other unknown nodes’ localization.

All of these algorithms mentioned above assume the nodes drift freely with ocean currents and need the presence of beacon nodes prior to localization. Moreover, the number of beacon nodes often increases with the network scale. This increases both the network cost and the difficulties of network recycling and maintenance. In [21], the authors assume nodes drift in a restricted manner and propose a localization algorithm without beacon nodes, called AFLA. The algorithm takes advantage of the geometrical relationship of three adjacent nodes and forms six equations to figure out their locations. However, AFLA does not take into account the noises varying with distances and has a high computational cost due to direct search in the solution space, which makes it inapplicable for UASN localization. In this paper, we adopt the similar network architecture and propose the MAP-PSO algorithm to solve the problems of varying noises and computation complexity.

## 3. Network Model and Problem Definition

In this section, we present the network model, analyze the drifting characteristic of underwater nodes and present formal definition of the localization problem.

### 3.1. Network Model

We consider an UASN that consists of a number of nodes deployed at the surveillance area. To reduce the network cost, each node is low-complexity with constrained energy and limited computational ability. Due to the intrinsic fluid property of underwater environments, the nodes move continuously with ocean currents. This requires the localization should be accomplished in a short time, otherwise the estimated locations will become obsolete as the nodes move to new locations. Hence, it is necessary to design a fast and energy-efficient algorithm to provide real-time localization in this resource-constrained network.

**Figure 1:**
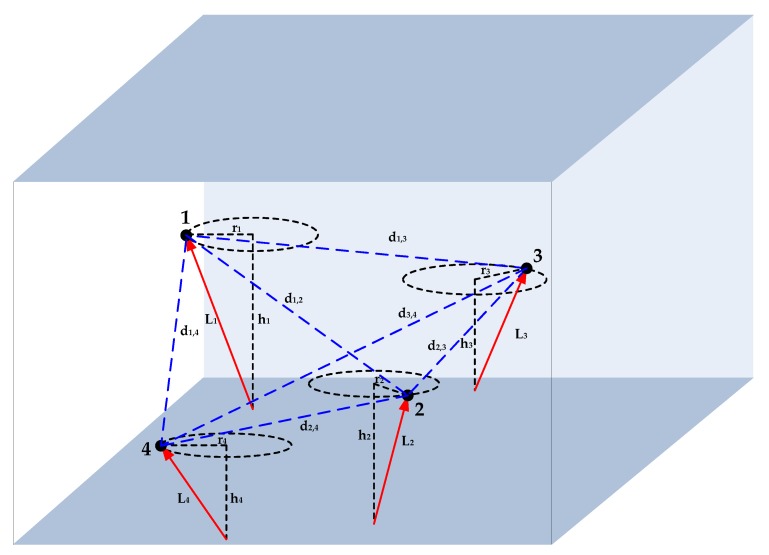
Network model.

As a consequence of the continuous mobility, some nodes may drift out of the deployment area, which increases the difficulties of network recycling and maintenance. Aiming at this problem, we follow a drifting-restricted UASN. Its network architecture is shown in Figure 1. Each node is linked to an anchor point by a cable and thus the movement of each node is confined in a local area. The locations of anchor points and the length of cables are known beforehand. Take node *i* for example, let $L_{i}$ denotes the length of its cable and $A_{i}$ denotes the location of its anchor point. In practical scenarios, the cable length may vary with surveillance requirements (e.g., tens of meters in nearshore areas [33] and hundreds of meters in offshore areas [34,35]). At the deployment phase, to prevent the cables of different nodes from twisting together, the distance between the anchor points of nodes *i* and *j* should be longer than the sum of the length of their cables:

(1) $$\left|\right|A_{i}-A_{j}{\left|\right|}_{2}\geq L_{i}+L_{j}$$

To ensure the nodes float in the water all the time, the length of the cables should be less than the sea depth *H*. Due to the impact of tide rise and fall, the sea depth should satisfy:

$$H_{min}<H<H_{max}$$

where $H_{min}$ denotes the sea depth of the highest tide and $H_{max}$ denotes the sea depth of the lowest tide. Therefore, the length of the cables satisfies:

$$0<{\left\{L_{i}\right\}}_{i=1}^{N}<H_{min}$$

At the same time, due to the tension of the cable, the depth of each node satisfies:

$$0<h_{i}<L_{i}$$

Generally, the movement of the nodes is controlled by joint forces from ocean current, water buoyancy and the cables. The buoyancy of each node is related to its volume, the water density and the acceleration of gravity. The water density is further influenced by ocean temperature and salinity. Hence the buoyancy of each node is a constant value in a certain spatial and temporal extent due to slow change of ocean temperature and salinity. The force of ocean current is mainly influenced by the current speed.

At a specific depth, the three forces reach a balance state. The node can drift on the plane of this depth and move along a circle centered at its anchor point. According to Pythagorean theorem, we have the following equation for node *i*:

(2) $$h_{i}^{2}+r_{i}^{2}=L_{i}^{2}$$

where $r_{i}$ denotes the radius of its movement circle. Ideally, if we assume ocean current is infinitesimal, the forces from water buoyancy and the cables reach balance in the vertical direction, then we have $h_{i}=L_{i}$ and $r_{i}=0$; on the contrary, if we assume ocean current is infinite, we obtain $h_{i}=0$ and $r_{i}=L_{i}$. Hence, according to different current speed, the movement radius *r* is $0<r_{i}<L_{i}$.

In practice, the depth $h_{i}$ can be obtained by equipping with a cheap pressure sensor. According to Equation (2), the radius $r_{i}$ can be calculated as $r_{i}=\sqrt{L_{i}^{2}-h_{i}^{2}}$. Therefore, the location of node *i* is constrained on a circle centered at the anchor point $A_{i}$ with the radius $r_{i}$. This indicates that the surveillance coverage will increase with the cable length and the node depth. This priori localization information hidden in the network model, as we will see later, can simplify the search space of localization solutions and reduce the localization time greatly.

To exploit the spatial relationship between nodes for localization, a node communicates with its neighbor nodes and measures the distances to them by multiplying the propagation delay by the acoustic speed. Most researches adopt one-way TOA method for distance estimation [5]. However, this method requires time synchronization between nodes, which is non-trivial in the harsh underwater environments. Alternatively, we adopt two-way TOA method for two reasons. One reason is that two-way TOA method can eliminate the clock offset between nodes and thus time synchronization is not requisite. The other reason is that the acknowledgement mechanism is commonly used in UASN MAC layer due to the inherent unreliability of underwater acoustic channel. Even if we adopt one-way TOA method, each packet needs an acknowledgement to guarantee successful packet transmission, which is similar with two-way TOA method. There have been multiple MAC protocols [36,37] for arranging packet transmissions and solve packet collisions, which is out of the scope of this paper. Herein, we take two nodes A and B as an example to illustrate the packet exchanges of two-way TOA method. A sends a ranging packet at time instant $t_{1}$. B receives the packet at time instant $t_{2}$ and responds with an acknowledgement at time instant $t_{3}$. Then A receives the response at time instant $t_{4}$, the propagation delay can be calculated as

(3) $$t_{p}=\frac{t_{4}+t_{2}-t_{1}-t_{3}}{2}$$

After that, the distance between A and B can be estimated as

(4) $$d=t_{p}\times c$$

where *c* represents the speed of acoustic signals. In each packet exchange, a node sends a packet at the power level $P_{tx}$ and receives a packet at the power level $P_{rx}$. Let $L_{P}$ and $L_{A}$ represent the size of ranging packet and acknowledgement packet. Hence, the energy consumption of the transmitting node is

$$E_{tx}=P_{tx}\times \frac{L_{P}}{R}+P_{rx}\times \frac{L_{A}}{R}$$

where R denotes the data rate. The energy consumption of the receiving node is

$$E_{rx}=P_{rx}\times \frac{L_{P}}{R}+P_{tx}\times \frac{L_{A}}{R}$$

Similarly, we can estimate the distances between a node and all of its neighbor nodes and calculate the overall energy consumption. Based on the priori localization information and spatial relationship between nodes, our purpose is to accurately estimate the locations of all the nodes with low computational and communication costs as fast as possible. Next we will present formal definition of the localization problem.

### 3.2. Problem Definition

Suppose there are *N* nodes in the whole network and each node needs to get its location periodically. We define *T* as the localization period in which each node needs to estimate its location. Note that, the localization period can be tuned according to practical requirements (e.g., in a UASN that observing ocean phenomena, it can be set to be equal to an hour [1]). For simplicity, the superscript for the n-th localization period is suppressed in what follows. The node *i* moves at the circle centered at the anchor point $A_{i}$ with the radius $r_{i}$. Therefore, its two-dimensional location can be represented as

(5) $$\left\{\begin{array}{c} x_{i}\;=x_{A}^{\;i}+r_{i}\cos\theta_{i}\\ y_{i}\;=y_{A}^{\;i}+r_{i}\sin\theta_{i} \end{array}\right.$$

where $\left(x_{i},y_{i}\right)$ denotes the locations of node *i* in the X- and Y-axes directions, $\left(x_{A}^{\;i},y_{A}^{\;i}\right)$ denotes the locations of anchor point $A_{i}$ in the X- and Y-axes directions, and θ denotes the azimuth angle between the X-axis and the line that connects the anchor point and the node. On this basis, we aim to find *M* nodes to form a node cluster S. The number *M* should be more than 3 (e.g., M=4 in Figure 1) so that a localization polygon can be constructed. Moreover, these nodes should be mutually neighbor nodes. Hence, for any two nodes *i* and *j* in the cluster, their locations satisfy the equation

$$\left|\right|X_{i}-X_{j}{\left|\right|}_{2}\leq min\left(C_{i},C_{j}\right)$$

where $X_{i}={\left(x_{i},y_{i},h_{i}\right)}^{T}$ is the three-dimensional location of node *i*, and $C_{i}$ denotes the communication radius of node *i*. The distance estimation $z_{i,j}$ between the two nodes can be obtained using Equations (3) and (4). In the cluster, there are totally M(M−1)/2 node pairs and each pair has its corresponding distance estimation. Hence the measurements of the cluster S can be represented as $Z=\{z_{i,j}|\forall i,j=1,2,\ldots ,M,i<j\}$. Similarly, we can find all the other clusters in the network and obtain their corresponding measurements.

For each cluster, we now have represented the two-dimensional location of the nodes and obtained the distance estimation between any two nodes. Given these data, the localization problem is to fuse them using a proper model and figure out the locations of all the cluster nodes. Specifically, our purpose is to minimize the sum of the squares of the errors between estimated distances and real distances. The objective function can be written as

(6) $$\underset{X_{1},X_{2},\ldots ,X_{M}}{\arg\min}\sum_{z_{i,j}\in Z}\left|\right|z_{i,j}-\left|\right|X_{i}-X_{j}{\left|||\right|}^{2}$$

The locations of *M* nodes can be resolved using efficient optimization methods. The whole localization process lasts until all the clusters have been localized. Next, we will present the algorithm that can fast localize the nodes without ambiguity.

## 4. MAP-PSO Design

In this section, we present MAP-PSO, a novel localization scheme without the presence of beacon nodes for DR-UASNs. The whole localization process consists of MAP estimation and PSO localization. In the following, we describe the details of our MAP-PSO algorithm starting from the MAP estimation step and followed by the PSO localization step.

### 4.1. MAP Estimation

Recall the localization problem defined in Equation (6), we didn’t take the noise into account, which may incur large errors of location estimations. Here, we consider two types of noise. One is that every node may not accurately move along its corresponding circle due to the error introduced in the practical deployments, such as the displacement of anchor points, the error of cable length, the volume of nodes, etc. The other is the distance measurements between nodes may deviate from real distances due to complex underwater environment factors, including non-straight propagation of acoustic signals, varying acoustic speed and tiny propagation time errors from clock skew.

To characterize the nodes’ mobility patterns, we assume the location of each node is randomly distributed around its corresponding movement circle, which can be represented as

(7) $$\left\{\begin{array}{c} x_{i}\;=x_{A}^{\;i}+r_{i}\cos\theta_{i}+w_{x}^{i}\\ y_{i}\;=y_{A}^{\;i}+r_{i}\sin\theta_{i}+w_{y}^{i} \end{array}\right.$$

where $w_{x}^{i}$ and $w_{y}^{i}$ denote the noises of the location of node *i* along X- and Y-axes, and follow a Gaussian distribution with zero mean and precision Λ, that is, $w_{x}^{i}∼N\left(0,\Lambda \right)$ and $w_{y}^{i}∼N\left(0,\Lambda \right)$. This implies that the noises of different nodes are assumed to be identically distributed. Then, we transform Equation (7) to its vector form and obtain the following formula

(8) $$x_{i}={\hat{x}}_{i}+w_{i}$$

where $x_{i}=\left(x_{i},y_{i}\right)$ denotes the two-dimensional location of node *i*, ${\hat{x}}_{i}={\left(x_{A}^{\;i}+r_{i}\cos\theta_{i},y_{A}^{\;i}+r_{i}\sin\theta_{i}\right)}^{T}$ and $w_{i}={\left(w_{x}^{i},w_{y}^{i}\right)}^{T}$. For simplicity, we assume $w_{x}^{i}$ and $w_{y}^{i}$ are uncorrelated with each other. Then the noise $w_{i}$ is a Gaussian variable with zero mean and the precision matrix ΛI, where I is the identity matrix. Hence, the location of node *i* follows a joint Gaussian distribution with mean ${\hat{x}}_{i}$ and precision ΛI:

(9) $$p\left(x_{i}\right)=N\left(x_{i}|{\hat{x}}_{i},\Lambda I\right)$$

Here, the depth $h_{i}$ is not considered because it can be measured beforehand by a pressure sensor. The uncertainty of the location $x_{i}$ and radius $r_{i}$ due to inaccuracy of depth measurements can be accommodated by the Gaussian distribution in Equation (9). It is important to note that this probability distribution is extracted from DR-UASN architecture, which can be treated as the prior knowledge of MAP estimation.

We now discuss how to model the noises in distance measurements. In [10], it has been found that the TOA error increases with real distance due to non-straight propagation. Hence, it is reasonable to assume that the ranging error caused by noise increases linearly with the real distance. Here, we use both additive and multiplicative noises [38] to model the distance measurements:

(10) $$z_{i,j}=\left(1+\alpha_{i,j}\right)d_{i,j}+\beta_{i,j}=d_{i,j}+\epsilon_{i,j}$$

where $d_{i,j}=\left|\right|X_{i}-X_{j}\left|\right|$ denotes the real distance between node *i* and *j*, $\alpha_{i,j}$ is the multiplicative noise that follows a Gaussian distribution with mean $\mu_{\alpha }$ and precision $\lambda_{\alpha }$, that is, $\alpha_{i,j}∼N\left(\mu_{\alpha },\lambda_{\alpha }\right)$, and $\beta_{i,j}$ is the additive noise that follows a Gaussian distribution with zero mean and precision $\lambda_{\beta }$, namely, $\beta_{i,j}∼N\left(0,\lambda_{\beta }\right)$. For simplicity, we assume that these two types of noises are uncorrelated with each other. The total noise can be denoted by $\epsilon_{i,j}=\alpha_{i,j}d_{i,j}+\beta_{i,j}$, which is also a Gaussian variable with mean $\mu_{i,j}=\mu_{\alpha }d_{i,j}$ and precision $\lambda_{i,j}={\left(\lambda_{\alpha }^{-1}d_{i,j}^{2}+\lambda_{\beta }^{-1}\right)}^{-1}$. Then, the conditional probability distribution of $z_{i,j}$ over $x_{i}$ and $x_{j}$ can be written as

(11) $$p\left(z_{i,j}|x_{i},x_{j}\right)=\frac{1}{\sqrt{2\pi }}\lambda_{i,j}^{\frac{1}{2}}\exp\left\{-\frac{1}{2}\lambda_{i,j}{\left(z_{i,j}-d_{i,j}-\mu_{\alpha }d_{i,j}\right)}^{2}\right\}$$

For all the distance measurements Z in the cluster S, the likelihood function of the locations $X={\left\{x_{i}\right\}}_{i=1}^{M}$ can be written as

(12) $$p\left(Z|X\right)=\prod_{z_{i,j}\in Z}p\left(z_{i,j}|x_{i},x_{j}\right)$$

Based on Equation (9), the priori probability distribution of the locations X is given by

(13) $$p\left(X\right)=\prod_{i=1}^{M}p\left(x_{i}\right)$$

Using the Bayesian theorem, the posterior probability distribution of X given the measurements Z can be derived as

(14) $$\begin{array}{cc} p(X|Z) & =p(Z|X)p(X)\\ & =\prod_{z_{i,j}\in Z}\ln p\left(z_{i,j}|x_{i},x_{j}\right)\prod_{i=1}^{M}\ln p\left(x_{i}\right) \end{array}$$

Then the locations X can be determined by maximizing the posterior distribution p(X|Z). Taking the logarithm of p(X|Z), we have

(15) $$\begin{array}{cc} \ln p(X|Z) & =\sum_{z_{i,j}\in Z}\ln p\left(z_{i,j}|x_{i},x_{j}\right)+\sum_{i=1}^{M}\ln p\left(x_{i}\right) \end{array}$$

Substituting Equations (9) and (11) into Equation (15), the maximum of the posterior probability is given by the minimum of

(16) $$\sum_{z_{i,j}\in Z}\hat{\lambda_{i,j}}{\left(z_{i,j}-d_{i,j}-\mu_{\alpha }d_{i,j}\right)}^{2}+\sum_{i=1}^{M}\Lambda I{\left(x_{i}-\hat{x_{i}}\right)}^{2}$$

where the irrelevant terms with the unknown locations X terms are omitted. Note that, considering $\lambda_{i,j}$ depends on the real distance $d_{i,j}$ that are determined by the unknown variable X, we have replaced $\lambda_{i,j}$ with $\hat{\lambda_{i,j}}={\left(\lambda_{\alpha }^{-1}z_{i,j}^{2}+\lambda_{\beta }^{-1}\right)}^{-1}$ under the assumption of the small deviations between $z_{i,j}$ and $d_{i,j}$.

Now let us analyze the ratio $\hat{\lambda_{i,j}}$ to $\hat{\lambda_{k,l}}$ ($z_{i,j},z_{k,l}\in Z$):

$$\begin{array}{cc} \frac{\hat{\lambda_{i,j}}}{\hat{\lambda_{k,l}}} & =\frac{{\left(\lambda_{\alpha }^{-1}z_{i,j}^{2}+\lambda_{\beta }^{-1}\right)}^{-1}}{{\left(\lambda_{\alpha }^{-1}z_{k,l}^{2}+\lambda_{\beta }^{-1}\right)}^{-1}} \end{array}$$

Multiplying both numerator and denominator with $\lambda_{\beta }^{-1}$, we get

$$\begin{array}{cc} \frac{\hat{\lambda_{i,j}}}{\hat{\lambda_{k,l}}} & =\frac{{\left(\lambda_{\alpha }^{-1}\lambda_{\beta }z_{i,j}^{2}+1\right)}^{-1}}{{\left(\lambda_{\alpha }^{-1}\lambda_{\beta }z_{k,l}^{2}+1\right)}^{-1}} \end{array}$$

In the simulations, we have assigned approximate values to $\lambda_{\alpha }$ and $\lambda_{\beta }$, that is, $\lambda_{\alpha }^{-1}\lambda_{\beta }\approx 1$. Taking the assumption that $z_{i,j}\gg 1$, which is reasonable for DR-UASNs, the above equation can be reduced as

$$\begin{array}{cc} \frac{\hat{\lambda_{i,j}}}{\hat{\lambda_{k,l}}} & \approx \frac{z_{i,j}^{-2}}{z_{k,l}^{-2}} \end{array}$$

Then we can replace $\hat{\lambda_{i,j}}$ with $z_{i,j}^{-2}$ in Equation (16) and execute equal operations to the second sum term. The objective function can be rewritten as:

(17) $$\min\sum_{z_{i,j}\in Z}z_{i,j}^{-2}{\left(z_{i,j}-d_{i,j}-\mu_{\alpha }d_{i,j}\right)}^{2}+\sum_{i=1}^{M}\delta I{\left(x_{i}-\hat{x_{i}}\right)}^{2}$$

where $\delta =\lambda_{\alpha }^{-1}\Lambda$. This function has two implications. On one hand, for the first sum term, each square error term has its own weight that is inversely proportional to the square of the distance measurement. Thus the location variables X tend to determine their values that fit the square terms with high weights better. On the other hand, the parameter δ can be regarded as a penalty factor between the priori knowledge and the likelihood information. Assigning a large value to δ propels the location of each node to be at its movement circle accurately; on the contrary, if δ is assigned a small value, each node is allowed to deviate its movement circle to certain extent and its location is more determined by the likelihood information.

### 4.2. PSO Localization

The minimization problem of the objective function in Equation (17) has no analytic solutions due to it is nonlinear function of the location variable X. Traditional optimization methods such as gradient descent easily fall into local optimum and have low convergence speed. In this paper, we resort to PSO method to solve this minimization problem. It uses a population of particles to represent candidate solutions in the search space, moves these particles to local and global best solutions iteratively, and then finds the particle that fit the objective function best. The PSO method can efficiently escape from local optimum by considering local and global views simultaneously. Moreover, we propose the bound constraint mechanism to improve the convergence speed greatly. Next, we describe the PSO procedure in detail.

In Equation (17), besides the location variable X, the optimization variables also include $\mu_{\alpha }$. Hence there are totally 2M+1 variables, which can be denoted as $\left\{{\left\{x_{i}\right\}}_{i=1}^{M},{\left\{y_{i}\right\}}_{i=1}^{M},\mu_{\alpha }\right\}$. We assume that there are $N_{P}$ particles initialized, that is, ${\left\{P_{k}\right\}}_{k=1}^{N_{P}}$. Each of the particles represents an instance of the variable set. To reduce the search space, we initialize the position of the particle as follows:

(18) $$P_{k}^{1}=\left[\begin{array}{c} x_{A}^{1}-r_{1}+rand\times 2r_{1}\\ y_{A}^{1}-r_{1}+rand\times 2r_{1}\\ .\\ .\\ .\\ x_{A}^{M}-r_{M}+rand\times 2r_{M}\\ y_{A}^{M}-r_{M}+rand\times 2r_{M}\\ \mu_{\alpha }^{L}+rand\times \left(\mu_{\alpha }^{U}-\mu_{\alpha }^{L}\right) \end{array}\right]$$

where $\mu_{\alpha }^{L}$ and $\mu_{\alpha }^{U}$ represent the lower and upper bound of $\mu_{\alpha }$ respectively, and rand is a random number in the range of [0,1]. Note that, $\mu_{\alpha }^{L}$ and $\mu_{\alpha }^{U}$ can be determined based on the knowledge of the surveillance area or estimated by running multiple tests between two nodes with known locations.

Once the particles are initialized, they move to new positions with a certain speed in the search space. By calculating the value of the objective function, they can find the local best position pBest. Then the particles communicate with each other to know the global best position gBest. After that, the velocity and the position of each particle is updated as follows

(19) $$v_{k}^{t+1}=\varpi \cdot v_{k}^{t}+c_{1}\cdot rand\cdot \left(pBest_{k}^{t}-P_{k}^{t}\right)+c_{2}\cdot rand\cdot \left(gBest^{t}-P_{k}^{t}\right)$$

(20) $$P_{k}^{t+1}=P_{k}^{t}+v_{k}^{t+1}$$

where ϖ is inertial weight, $c_{1}$ is cognitive acceleration factor, $c_{2}$ is social acceleration factor, rand is a random number in the range of [0,1], $pBest_{k}^{t}$ denotes the best solution found by particle *k* at iteration *t*, $gBest^{t}$ denotes the best solution found in the particle swarm at iteration *t*, $v_{k}^{t}$ and $P_{k}^{t}$ denote the velocity and position of particle *k* at iteration *t*, respectively. The parameter ϖ is critical to keep balance between local and global search. At the later phase of optimization, to avoid the oscillation phenomenon and improve the local search capability, the linear decreasing inertia weight [39] is commonly adopted to slow the particles over time. The inertial weight is updated as follows

(21) $$\varpi =\varpi_{max}-\frac{\varpi_{max}-\varpi_{min}}{t_{max}}\times t$$

where $\varpi_{max}$ and $\varpi_{min}$ are the maximum and minimum weights respectively, and $t_{max}$ is the maximum iteration number.

The optimization repeats the operations in Equations (19)–(21) until the termination condition is satisfied. There are two termination conditions in our method. One is the relative change of the best objective function value over $t_{l}$ iterations is less than a predefined threshold ρ; the other is the current iteration has reach the maximum iteration number, that is, $t=t_{max}$.

**Figure 2:**
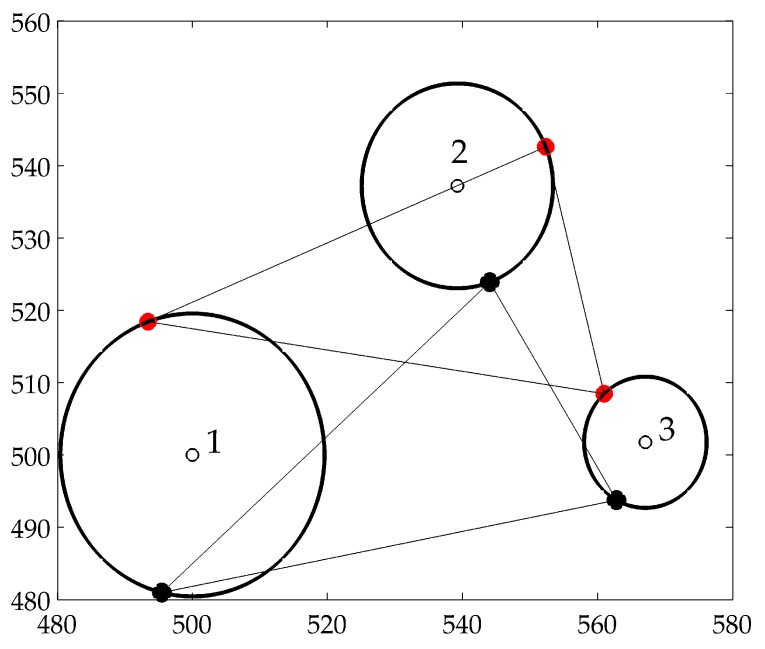
Localization ambiguity.

In the above optimization process, there exist two problems that limit the localization accuracy and the convergence speed. First, a cluster with a small *M* is prone to localization ambiguity. As shown in Figure 2, three black solid circles are the real locations of nodes 1, 2 and 3, whereas the optimization gives the estimated locations as three red solid circles, resulting in low localization accuracy. This is mainly because that the distance measurements generated by few nodes have little constraint on the nodes’ locations. Second, although we have bounded the position of each particle in the initialization phase, the optimizations of many clusters still have low convergence speed, especially in a high-dimensional space (a large *M*).

To solve the first problem, we propose a novel reference selection mechanism to eliminate the ambiguity in localization. In practice, different clusters may overlap, that is, for two clusters $S_{1}$ and $S_{2}$, part of their nodes are the same, $S_{1}\cap S_{2}\neq \emptyset$. If $S_{1}$ is first optimized, the common nodes that are localized with high accuracy can be regarded as reference nodes to eliminate the localization ambiguity in the optimization process of $S_{2}$. Traditional localization schemes mainly use the confidence value to indicate the localization accuracy, which can be calculated as

(22) $$\eta =1-\frac{\sum_{i}\left|{\left(u-u_{i}\right)}^{2}+{\left(v-v_{i}\right)}^{2}+{\left(w-w_{i}\right)}^{2}-l_{i}^{2}\right|}{\sum_{i}{\left(u-u_{i}\right)}^{2}+{\left(v-v_{i}\right)}^{2}+{\left(w-w_{i}\right)}^{2}}$$

where (u,v,w) is the estimated location of the unknown node, $\left(u_{i},v_{i},w_{i}\right)$ is the location of reference node *i* and $l_{i}$ is the distance measurement between the unknown node and reference node *i*. However, this confidence mechanism does not hold true in our method. Take the nodes in Figure 2 for example, even though the estimated locations marked red has large deviations from the real locations marked black, the nodes 1, 2 and 3 have high confidence values because the distances based on the estimated locations fit the distance measurements well. Alternatively, we propose to select the reference nodes according to localization stability. This approach is simple, yet effective. Specifically, we define the sliding window with length set to $T_{w}$ that is an integer multiple of *T*, i.e., $T_{w}=B\times T$. A node *i* can act as a reference node when the following two conditions are satisfied: (1) the node should be localized in every localization period of a sliding window; (2) the change of the estimated locations of the node in any two consecutive localization periods should be below the maximum movement distance, $\left|\right|X_{i}^{n}-X_{i}^{n+1}\left|\right|<V_{max}\times T$, where $X_{i}^{n}$ and $X_{i}^{n+1}$ denote the estimated location of the node at two consecutive localization period, $V_{max}$ denotes the maximum velocity of nodes.

Aiming at the second problem, we propose to further confine the particles’ positions on the basis of their values initialized in Equation (18). As we have discussed above, a reference node has strong localization stability because its estimated locations have little changes in a sliding window. Then we can use this characteristic to confine the search space of the reference nodes. Let $X_{j}^{m}$ and $\theta_{j}^{m}$ denote the location and azimuth angle of the reference node *j* at the localization period *m*, respectively. Our approach is to set the tight bound of the location of the reference node at the localization period m+1 and thus speed up the convergence of the optimization. We assume the cable linked to the reference node is always tense. The path that the reference node moves along is actually an arc, and its length should be less than the maximum movement distance $V_{max}\times T$. Accordingly, the maximum movement angle is calculated as

(23) $$\theta_{j}^{max}=\frac{V_{max}\times T}{\pi \times r_{j}}$$

Then the azimuth angle $\theta_{j}^{m+1}$ belongs to the range $\left[\theta_{j}^{LB},\theta_{j}^{UB}\right]$, where the lower bound $\theta_{j}^{LB}=\theta_{j}^{m}-\theta_{j}^{max}$ and the upper bound $\theta_{j}^{UB}=\theta_{j}^{m}+\theta_{j}^{max}$. According to Equation (5), the node locates at $\left(x_{A}^{\;j}+r_{j}\cos\theta_{j}^{LB},y_{A}^{\;j}+r_{j}\sin\theta_{j}^{LB}\right)$ and $\left(x_{A}^{\;j}+r_{j}\cos\theta_{j}^{UB},y_{A}^{\;j}+r_{j}\sin\theta_{j}^{UB}\right)$ for the lower and upper bound of the azimuth angle. Hence, for the reference node *j*, we can set the lower bound of its X- and Y-axes location as $min(x_{A}^{\;j}+r_{j}\cos\theta_{j}^{LB},x_{A}^{\;j}+r_{j}\cos\theta_{j}^{UB})$ and $min(y_{A}^{\;j}+r_{j}\sin\theta_{j}^{LB},y_{A}^{\;j}+r_{j}\sin\theta_{j}^{UB})$, and set the upper bound of its X- and Y-axes location as $max(x_{A}^{\;j}+r_{j}\cos\theta_{j}^{LB},x_{A}^{\;j}+r_{j}\cos\theta_{j}^{UB})$ and $max(y_{A}^{\;j}+r_{j}\sin\theta_{j}^{LB},y_{A}^{\;j}+r_{j}\sin\theta_{j}^{UB})$. This tight bound not only improves the convergence speed, but also propels the estimated locations of other nodes to their real locations.

## 5. Performance Evaluation

In this section, we evaluate the performance of the MAP-PSO algorithm using simulations.

### 5.1. Simulation Settings

In our simulations, 100 unknown nodes are deployed in a cubic region with the range 1000 m × 1000 m × 20 m. MAP-PSO does not need beacon nodes. Each node is linked to a fixed anchor point through a cable with the length 20 m. The anchor points of any two neighbor nodes should satisfy the Equation (1). The node depth is randomly distributed in the range [4,16], which ensures that every node floats underwater and has a proper monitoring coverage. Each node moves at the maximum velocity 10 m/s clockwise or anti-clockwise. The precision matrix of the noise of each node’s location is given by $\Lambda =50I_{2}$.

As for the distance measurements, a node uses two-way packets to communicate with its neighbor nodes and estimates the distance based on Equation (3) and (4). For energy consumption, we adopt the parameters given in [40]. The packet size is set to be $L_{P}=100$ bytes and the acknowledgement size is set to be $L_{A}=20$ bytes. To model LinkQuest UWM4000 underwater modem, the data rate, transmission and reception power are set as R=8.5 kbps, $P_{tx}=7$ W and $P_{rx}=0.8$ W, respectively. The communication range of all the nodes is set to be 150 m. The mean and precision of the multiplicative noise are set to be $\mu_{\alpha }=0.02$ and $\lambda_{\alpha }=60$, respectively, and the precision of the additive noise is set to be $\lambda_{\beta }=100$. Once the distance measurements for all the node pairs in a cluster are collected, the PSO method is used to resolve the nodes’ locations. The maximum iteration number is set to be 600. In each iteration, we set the number of the particles as $N_{P}=200$. Every particle updates itself according to Equations (19) and (20). The inertial weight is updated according to Equation (21), where the maximum and minimum weights are set to be $\varpi_{max}=0.9$ and $\varpi_{min}=0.4$, respectively. The cognitive and social acceleration factor are set to be $c_{1}=0.5$ and $c_{2}=1.25$, respectively. The optimization stops when the relative improvement of the best objective function value is less than $\rho =10^{-3}$ over $t_{l}=50$ iterations.

Every simulation lasts for 100 s. We set the localization period as T=1 s and use a sliding window with B=5 localization period, i.e., $T_{w}=5$ s. Every simulation is run 50 times to eliminate the impact of randomness. In the simulations, we consider three performance metrics, which are localization accuracy, localization coverage and localization time. Localization accuracy is defined as the average error between estimated locations and real locations of all the localized nodes, and the standard deviation of the localization error is calculated as

$$Std=\sqrt{\frac{1}{n}\sum_{i=1}^{n}{\left(e_{i}-\bar{e}\right)}^{2}}$$

where $e_{i}$ denotes the localization error of the i-th localized node and $\bar{e}$ denotes the average error of all the localized nodes. Localization coverage is defined as the proportion of the localized nodes to all nodes. Localization time is defined as the period that starts when all the distance measurements for a cluster are collected and ends when the locations of all the cluster nodes are obtained. Based on the above settings, MAP-PSO and AFAL are implemented using the MATLAB 2014a platform, on a computer with an Intel Core i3-6100 CPU 3.7 GHz and 8.0 GB RAM.

### 5.2. Localization Performance under Different Parameters

The performance of the MAP-PSO algorithm depends on many important parameters, including the penalty factor, the maximum iteration number, the particle number and the minimum cluster node number. We aim to determine the optimal values of these parameters by simulations.

#### 5.2.1. Impacts of the Penalty Factor

**Figure 3:**
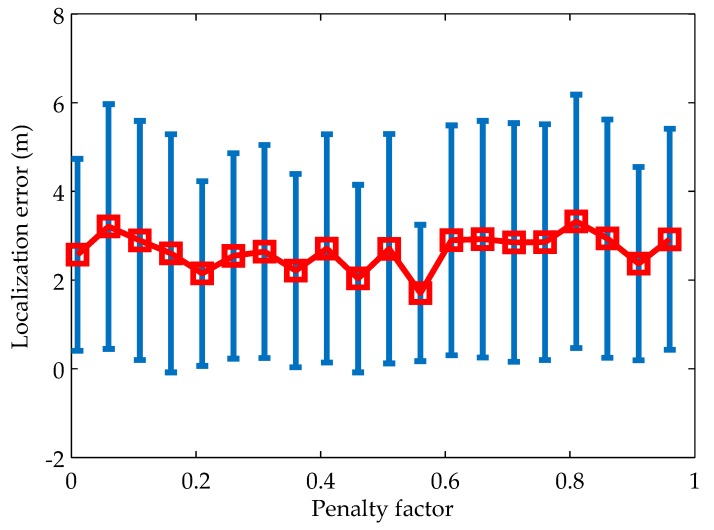
Impacts of the penalty factor on localization accuracy.

The penalty factor has an important impact on the localization accuracy. As we discussed above, assigning a proper value to the penalty factor will make a balance between the priori knowledge and the likelihood information. Therefore, it is necessary to determine the value of the penalty factor by simulations. Figure 3 shows the relationship between the localization error and the penalty factor. We can observe that there exists some critical value on the curve, where the algorithm has the minimum localization error and standard deviation. This implies that the algorithm has a strong robustness at this value. When the penalty factor is below this value, the algorithm overfits the noisy distance measurements and underfits the priori mobility pattern. On the contrary, if the penalty factor is above this value, the algorithm underfits the noisy distance measurements and overfits the priori mobility pattern. In both cases, the algorithm has larger localization error and standard deviation.

#### 5.2.2. Impacts of The Maximum Iteration Number

**Figure 4:**
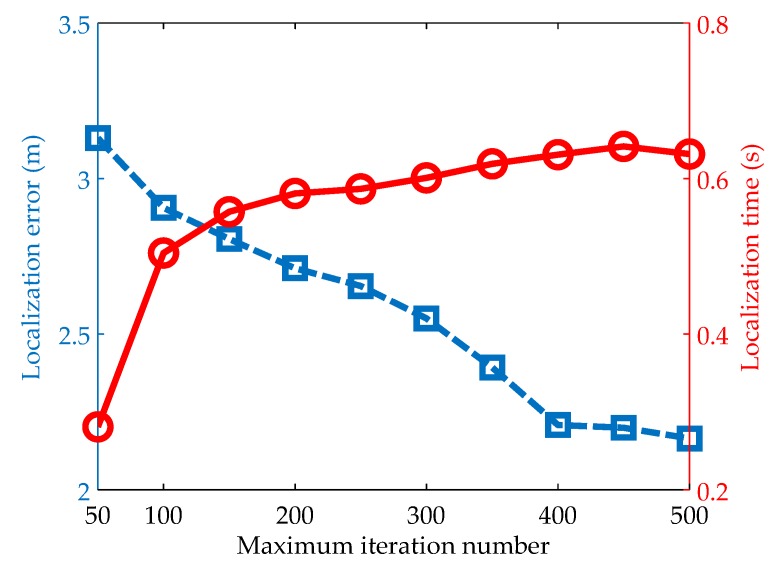
Impacts of the maximum iteration number on localization accuracy and time.

The maximum iteration number is another important factor that impacts the localization performance. On one hand, if the maximum iteration number is set to be too small, the optimization may stop too early to converge to the optimal solution, resulting in low localization accuracy; on the other hand, if setting the maximum iteration number to be large helps to improve the localization accuracy, the optimization needs more computational resource, which is infeasible for resource-constrained UASNs. By choosing the penalty factor as 0.56 according to Figure 3 and changing the maximum iteration number from 50 to 600, the localization error and time under different maximum iteration number are shown in Figure 4. We can see that with the increase of the maximum iteration number, the localization error decrease rapidly and the localization time increase rapidly in the first. When the maximum iteration number increases to some point (e.g., 400 in Figure 4), both the localization error and the localization time stabilize gradually. This is because the optimizations of most nodes reach the convergence in less than 400 iterations.

#### 5.2.3. Impacts of the Particle Number

**Figure 5:**
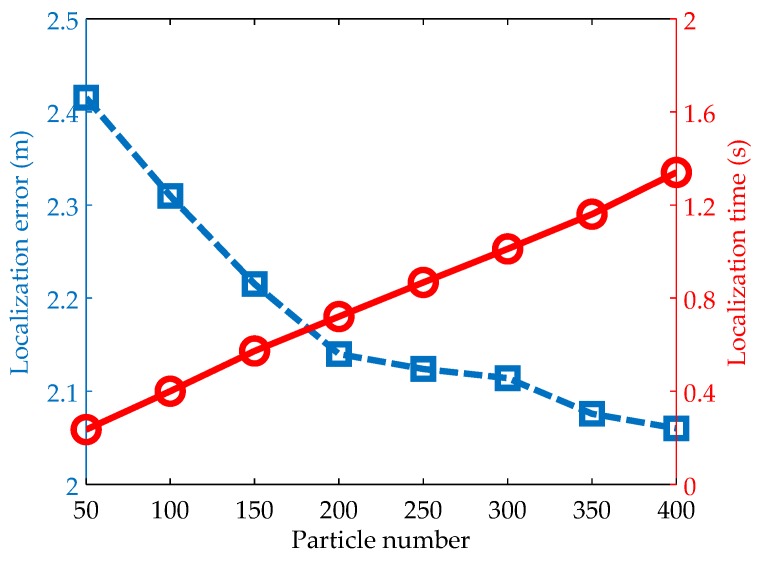
Impacts of the particle number on localization accuracy and time.

The number of particles is a critical parameter to determine the search ability of PSO localization. In theory, the search ability can be improved by simply increasing the particle number. However, a large number of particles have to be updated according to Equations (19) and (20), which leads to high computational cost. Hence, it is necessary to find a critical point, where a balance between the localization error and the localization time can be reached. By choosing the penalty factor and the maximum iteration number as 0.56 and 400 respectively, the simulation results using 50 to 400 particles are shown in Figure 5. From the figure, we have two observations: on one hand, the localization time increases linearly with the particle number; on the other hand, the localization error decreases rapidly with the increase of the particle number at the beginning. But with the further increase of the particle number (e.g., greater than 200 in Figure 5), the localization accuracy does not have clear improvement any longer. Therefore, it is reasonable to choose the particle number as 200, which makes a tradeoff between the localization error and the computational cost.

#### 5.2.4. Impacts of the Minimum Cluster Node Number

**Figure 6:**
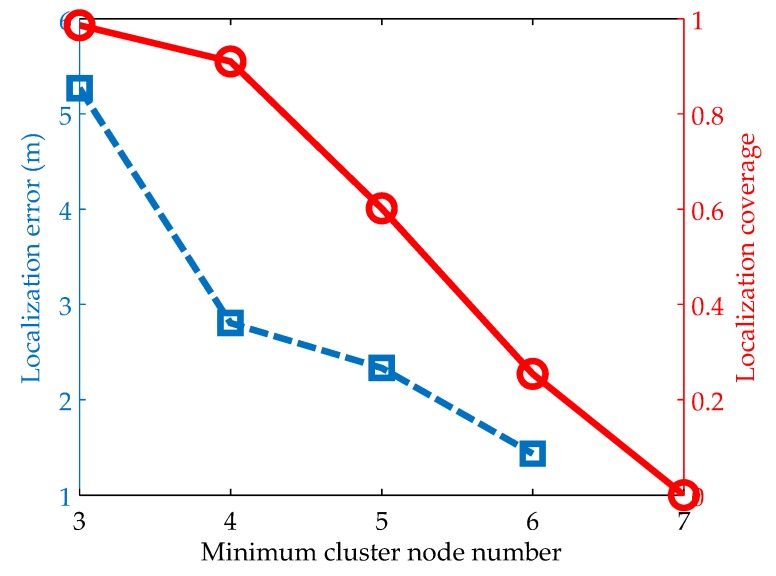
Impacts of the minimum cluster node number on localization accuracy and coverage.

In the above simulations, we ignore the localization coverage because it is rarely affected by the penalty factor, the iteration number and the particle number. We now evaluate the performance under the minimum cluster node number. This parameter means that a cluster can’t be localized if the number of its nodes is less than the minimum cluster node number. Figure 6 illustrates the results when the value of this parameter changes from 3 to 7. It is clear that when the minimum cluster node number is set to be small, the algorithm has low localization accuracy and high localization coverage. This is because most clusters can be localized when the number of their nodes is more than the minimum cluster node number. However, as we stated before, there exists the localization ambiguity due to that the polygon constructed by a small number of cluster nodes has little constraint on the nodes’ locations. As the minimum cluster node number grows, both the localization error and the localization coverage decreases rapidly. This is mainly because with the increase of the minimum cluster node number, the localization ambiguity can be eliminated gradually and few clusters satisfy the localization condition. Further, we can observe that there exists a turning point at the minimum cluster node number of 4. Below this value, the localization coverage does not decrease much and the localization accuracy is greatly improved. But above this value, the localization coverage decrease sharply and the localization accuracy has little improvement. In addition, the minimum cluster node number is closely related to the network density. As the network density grows, its value can be set to relatively large. In this way, the algorithm can achieve high localization accuracy and coverage simultaneously.

#### 5.2.5. Localization Results

**Figure 7:**
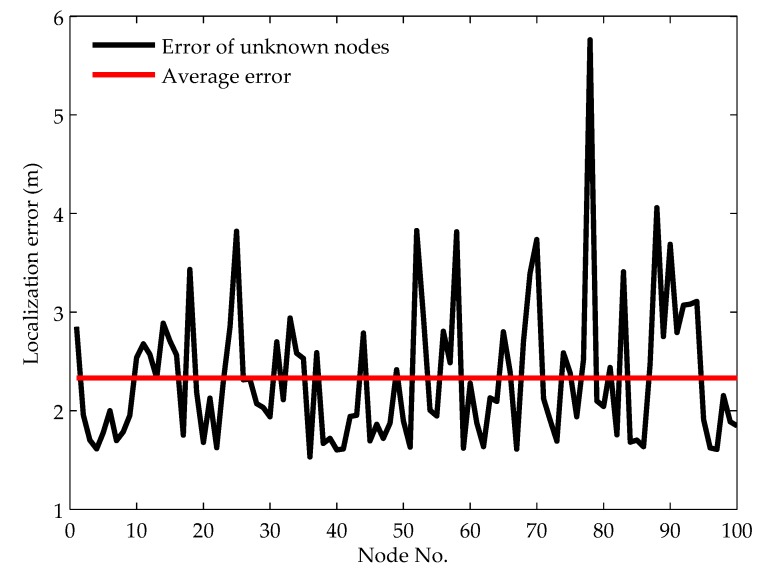
Localization error of each node.

By choosing the optimal values of the penalty factor, the maximum iteration number, the particle number and the minimum cluster node number, we set the node density as 8 by adjusting the communication range of the nodes as 160 m and evaluate the localization result of each node. Figure 7 shows the localization error of all the unknown nodes. The average localization error of all the unknown nodes is 2.33. The error of 59% unknown nodes is less than the average error and the error of 93% unknown nodes is less than 3.5 m, which means that most unknown nodes have high localization accuracy compared with the communication range. Furthermore, we note that the localization error of some unknown nodes has large values (such as nodes 25, 52, 88 and 78), which means the localization accuracy of these nodes is not very high. This is mainly because these nodes are localized when the number of nodes in their clusters is small, resulting in that the localization ambiguity is not eliminated completely. On the whole, the localization result indicates that the localization accuracy is satisfiable and the localization algorithm is robust to noisy underwater environments.

### 5.3. Performance Comparison with AFLA

In this section, we compare the localization accuracy and time of MAP-PSO with that of AFLA under different parameters, including the measurement noise level and the node density. The localization coverage is not evaluated because both algorithms have the same localization coverage under the two parameters. According to the above simulations, we choose the penalty factor, the maximum iteration number, the particle number and the minimum cluster node number as 0.56, 400, 200 and 4, respectively. The unique parameters of AFAL are set according to [21].

#### 5.3.1. Impacts of the Measurement Noise Level

**Figure 8:**
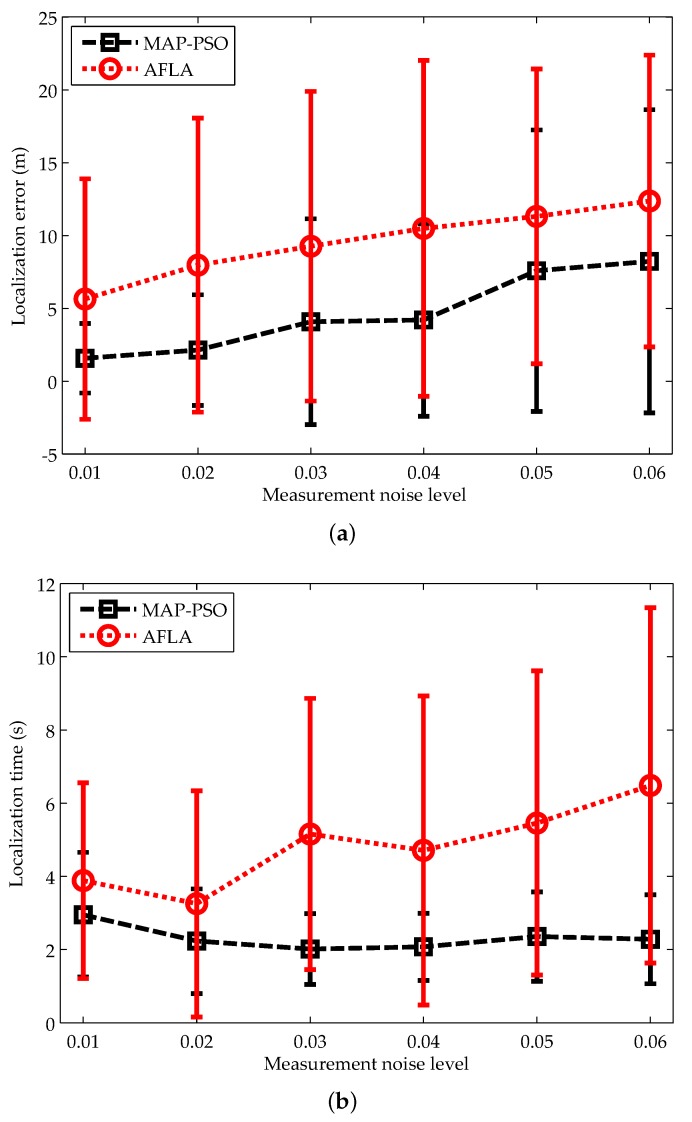
Impacts of the measurement noise level on: (**a**) localization accuracy; (**b**) localization time.

We first compare the performance of both algorithms under different measurement noise levels. As we stated before, the measurement noise is composed of the additive and multiplicative noises. Due to the additive noise is assumed to follow a Gaussian distribution with zero mean, it is reasonable to use the mean of the multiplicative noise to represent the measurement noise level. We change the mean of the multiplicative noise from 0.01 to 0.06. The results for localization accuracy and localization coverage are illustrated in Figure 8.

From Figure 8a, we can see that the localization errors of MAP-PSO and AFAL both increase with the measurement noise level. In comparison, MAP-PSO has higher localization accuracy and lower standard deviation. Meanwhile, as the measurement noise level grows, the standard deviation of AFAL increases faster than that of MAP-PSO, which implies that MAP-PSO is more robust to complex underwater noises. This is mainly because MAP-PSO fits the noisy measurements and the priori mobility pattern in a weighted way, while AFAL treats the two types of information equally. Especially, when the measurement noise is of high level, it is difficult for AFAL to find an exact solution that fit the two types of information well.

Figure 8b shows us the relationship between the localization time and the measurement noise level. It is obvious that MAP-PSO can complete the localization process in a shorter time and have lower standard deviation compared with AFAL. Furthermore, the localization time of AFAL increases rapidly with the measurement noise while that of MAP-PSO has no obvious change. This is because AFAL have to directly search the whole solution space when the measurement noise is of high level.

#### 5.3.2. Impacts of the Node Density

**Figure 9:**
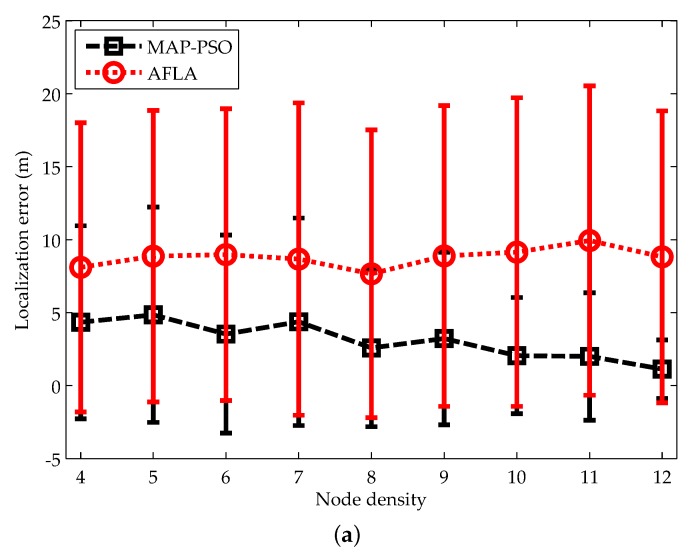

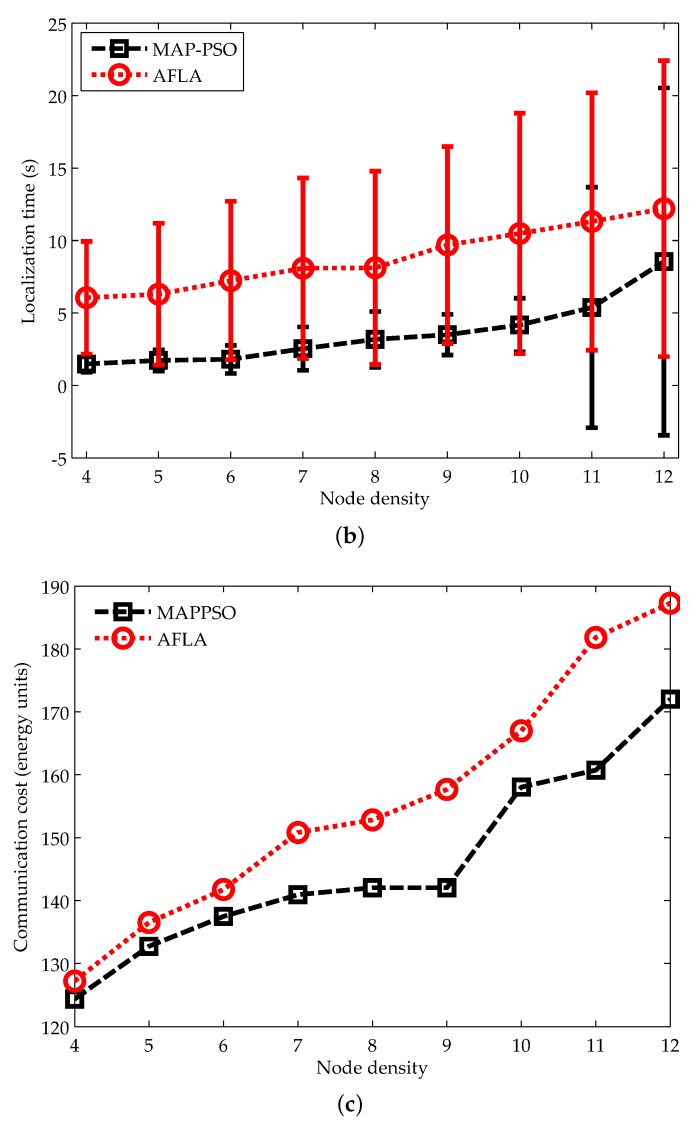
Impacts of the node density on: (**a**) localization accuracy; (**b**) localization time; (**c**) communication cost.

We next compare the performance of both algorithms under different node densities. Node density is defined as the expected number of nodes in a node’s neighborhood. We change the node density from 4 to 12 by adjusting the communication range of the nodes. The results for localization accuracy and localization coverage are illustrated in Figure 9.

As shown in Figure 9a, MAP-PSO achieves higher localization accuracy and lower standard deviation compared with AFAL. As the node density grows, the localization error of MAP-PSO decreases slowly, but that of AFAL has no obvious improvements. This is mainly because that the number of nodes in a cluster increases with the node density and thus more distance measurements can be generated. These measurements have strong constraints on the nodes’ locations, which can significantly relieve the localization ambiguity problem.

Figure 9b shows the relationship between the localization time and the node density. We can see that MAP-PSO takes less time for localization compared with AFAL. Besides, the localization time of MAP-PSO has the lower standard deviation. This indicates that each node has similar computational cost in our algorithm, which can efficiently prolong the network life. In addition, the localization time of MAP-PSO increases monotonically with the increase of the node density. This is because with the increase of the node density, the number of cluster nodes increases and the location variable is high-dimensional. By using a limited number of particles, searching the best solution in a high-dimensional space may need more iteration times.

Besides the localization accuracy and time, we evaluate the energy consumption under different node densities. For simplicity, we follow LDSN [22] to set the acknowledgement reception consume one unit of energy. According to the values of the parameters $L_{P}$, $L_{A}$, $P_{tx}$ and $P_{rx}$, we set the the ranging packet reception consume 5 units of energy, the acknowledgement transmission consume 9 units of energy and the ranging packet transimmsion consume 45 units of energy, respectively. As illustrated in Figure 9c, the energy consumption of the two algorithms increases with the node density. When the node density is relatively small, MAP-PSO and AFLA have similar energy consumption. This is because most clusters in MAP-PSO consist of three or four nodes in this case. As the node density grows, AFLA has more energy consumption than MAP-PSO. The reason may be that AFLA forms more clusters due to it adopts the three-node cluster localization.

## 6. Conclusions

In this paper, we considered the localization problem in UASNs where nodes are permanently moving with restrictions and measurement noises vary with the distances. We introduced a localization algorithm that uses the priori knowledge provided by nodes’ mobility patterns and the likelihood information offered by distance measurements. Under the Bayesian framework, the algorithm fuses the two types of information in a weighted way. In the localization phase, the algorithm fits the components in the localization objective function according to their weights. To improve localization accuracy and convergence speed, we further propose novel reference selection and bound constraint mechanisms. We evaluate the localization performance of our algorithm under different unique parameters. The simulation results give the suggestions of selecting proper values for these parameters. We also compare the localization performance with the benchmark AFAL algorithm. The simulation results show that our algorithm can use less localization time and energy consumption to achieve higher localization accuracy.

In the future, our work focuses on two aspects: on one hand, considering the depth measurements may be obtained with low accuracy from a cheap pressure sensor due to the variation of atmosphere and tides, we will study the accurate localization algorithm with coarse depth measurements; on the other hand, we will extend the proposed method to improve the localization performance by using temporal dependency between a node’s locations of consecutive time instants.
